# Transcriptomic responses to drought stress among natural populations provide insights into local adaptation of weeping forsythia

**DOI:** 10.1186/s12870-021-03075-6

**Published:** 2021-06-15

**Authors:** Yong Li, Long-Chen Shi, Nan-Cai Pei, Samuel A. Cushman, Yu-Tao Si

**Affiliations:** 1 Innovation Platform of Molecular Biology, College of Landscape and Art, Henan Agricultural University, Zhengzhou, China; 2 Research Institute of Tropical Forestry, Chinese Academy of Forestry, Guangzhou, China; 3 U.S. Forest Service, Rocky Mountain Research Station, 2500 S. Pine Knoll Dr., Flagstaff, AZ USA

**Keywords:** Climate change, Drought stress, Local adaptation, RNA-seq, Transcriptome, Weeping forsythia

## Abstract

**Background:**

Understanding the genetic mechanisms of local adaptation is an important emerging topic in molecular ecology and evolutionary biology.

**Results:**

Here, we identify the physiological changes and differential expression of genes among different weeping forsythia populations under drought stress in common garden experiments. Physiological results showed that HBWZ might have higher drought tolerance among four populations. RNA-seq results showed that significant differential expression in the genes responding to the synthesis of flavonoids, aromatic substances, aromatic amino acids, oxidation–reduction process, and transmembrane transport occured among four populations. By further reanalysis of results of previous studies, sequence differentiation was found in the genes related to the synthesis of aromatic substances among different weeping forsythia populations.

**Conclusions:**

Overall, our study supports the hypothesis that the dual differentiation in gene efficiency and expression increases among populations in response to heterogeneous environments and is an important evolutionary process of local adaptation. Here, we proposed a new working model of local adaptation of weeping forsythia populations under different intensities of drought stress, which provides new insights for understanding the genetic mechanisms of local adaptation for non-model species.

**Supplementary Information:**

The online version contains supplementary material available at 10.1186/s12870-021-03075-6.

## Background

Understanding the genetic mechanisms of local adaptation is an important topic in molecular ecology and evolutionary biology [1, 2]. Local adaptation refers adaptive genetic differentiation occurs among different populations in response to the local environment [3]. Adaptive genetic differentiation can produce differentiation in physiology, phenotype, and phenology to improve the fitness of species in the local environment [4]. Identifying the adaptive genetic divergence that improves species adaptability to local environments is critical to understand the mechanisms of local adaptation.

Although disentangling the genetic mechanisms of local adaptation is important for understanding adaptive evolution, the lack of genomic information of most species limits the species and systems in which local adaptation can be studied [5]. With the development of sequencing technology, especially third-generation sequencing technologies, such as PacBio RSII and Nanopore PromethION, the cost of genome sequencing has dropped significantly [6]. More species genomes have been published, which provides opportunities for us to explore the genetic mechanisms of local adaptation for non-model species.

Recently, there have been several examples of studies that successfully explored the mechanisms of local adaptation in a few species. For example, the natural populations of *Pterocarya stenoptera* are strongly differentiated in genes related to temperature, water, and light adaptation [7]. In addition, in the natural populations of *Corymbia calophylla,* adaptive differentiation occurs in the genes of temperature and precipitation [8]. Furthermore, in chocolate tree, the genes related to adaptive response to abiotic factors and pathogens have experienced adaptive differentiation [9]. These examples confirm that the genes undergo adaptive genetic differentiation to promote local fitness in different environments.

The adaptive differentiation among populations is the result of the genetic divergence in gene sequences. The genes identified by population genomics studies as possessing strong selective signals imply that the genes with sequence divergence among populations are likely different in coding regions. In recent common garden experiments, *Arabidopsis halleri* in metal-contaminated areas expressed adaptive differentiation in gene expression in response to high zinc stress, which indicates the differentiation of adaptive strategies among populations [10]. Under the same cultivation conditions, *Ricotia lunaria* showed significant differentiation of gene expression among different natural populations [11]. These studies indicate constitutive expression differences among different populations, even among those that are found in the same conditions or face to the same stresses.

These cases imply that in addition to the adaptive differentiation of gene sequence there may be adaptive differentiation of gene expression or other factors (i.e. methylation modification) [12] in the process of local adaptation. Thus, we propose as a new hypothesis that the dual differentiation in sequence differentiation and expression among populations in response to heterogeneous environments during local adaptation. This also brings a new issue, namely there are relationships between the gene sequence differentiation and those associated with gene expression differentiation?

Weeping forsythia (*Forsythia suspensa,* Oleaceae) is a dominant deciduous species in the warm-temperate region in China. It is also a famous medicinal plant, widely used in Chinese traditional medicine for treating colds with extracts from its fruits [13]. Due to the high market demand, weeping forsythia has become a new medicinal crop and has been cultivated in large areas in recent years. It may play an important role in treating COVID-19, given that it significantly mitigates the symptoms of pneumonia [14]. However, the study from Hu et al. [14] concerns patients with mild or ordinary COVID-19 symptoms, not severe COVID-19 symptom, and these results need to be further confirmed.

Although weeping forsythia is a drought-tolerant medicinal crop, long-term drought stress will affect its growth and fruit production. Recently, the genome sequence of weeping forsythia has been published, which provides opportunities for us to explore the genetic mechanisms of local adaptation [15]. Past studies using population genomics focused on detecting and quantifying adaptive differentiation in gene sequences in response to drought stress among different natural populations [15]. Whether there are differences in gene expression in response to drought stress among the natural populations of weeping forsythia, however, still required verification by common garden experiments.

In this study, the seeds from four natural populations of weeping forsythia were selected for common garden planting in a laboratory setting. Comparative transcriptome sequencing was used to examine the response to drought stress of the four weeping forsythia populations. The research objectives of this study were as follows: (i) whether gene expression differences in response to drought stress exist among the four weeping forsythia populations, and (ii) whether the genes with gene sequence differentiation and those with gene expression differentiation exist relationships.

## Results

### Physiological changes under drought treatment

Three physiological indexes under drought treatment were determined in this study (Table 1). After drought treatment, proline (Pro) content in Wulaofeng, Shanxi (SXWL) population increased by 25.3%, which was the most significant change among the four populations. For soluble sugar (SS), HBWZ is the population with the most significant growth (68.2%) after drought treatment. Although SS in Wuzhi Mountains, Hebei (HBWZ) and Pro in SXWL are not the highest in absolute content among the four populations, they are the most significant increase. Malondialdehyde (MDA) content in SXWL, Shaanxi Hua Mt. (SXHM), and Shaanxi Laojun Mt. (SXLJ) increased significantly after drought treatment, which increased 33.3, 41.2, and 42.9%, respectively. However, MDA in HBWZ did not change significantly after drought treatment, which indicated that drought stress did not cause peroxidation and damage to membrane system of HBWZ population samples. According to the rangeability of three indexes before and after drought treatment, HBWZ might show better resistance.

**Table 1:** Physiological indexes of four populations in weeping forsythia. The letters indicates the significance among four populations

Population	Pro content(μg∙g^−1^FW)		SS content(mg∙g^−1^FW)		MDA content (μmol∙g^−1^FW)	
	80%SWC ± SE	20%SWC ± SE	80%SWC ± SE	20%SWC ± SE	80%SWC ± SE	20%SWC ± SE
HBWZ	32.895 ± 2.263^b^	34.830 ± 4.514^ab^	8.001 ± 1.548^abc^	13.460 ± 3.902^a^	13.728 ± 1.089^ab^	11.841 ± 1.620^ab^
SXWL	44.012 ± 11.511^ab^	55.165 ± 10.656^a^	11.446 ± 1.287ab	9.774 ± 0.187^abc^	8.829 ± 0.247^b^	11.765 ± 1.582^ab^
SXHM	47.927 ± 6.629^ab^	55.878 ± 7.287^a^	5.519 ± 0.274c	6.644 ± 0.364^bc^	13.542 ± 3.057^ab^	19.122 ± 5.254^a^
SXLJ	41.440 ± 5.776^ab^	28.862 ± 1.124^b^	9.568 ± 1.377	13.441 ± 3.134^abc^	12.060 ± 2.757^ab^	17.238 ± 2.007a

### Transcriptome sequencing

To reveal the differences of gene expression in response to drought stress among the four weeping forsythia populations, transcriptome sequencing was performed on the samples collected under 80 and 20% soil water content (SWC). Following quality filtering from the raw reads yielded by RNA sequencing, 686,315,499 clean reads were obtained for all 24 weeping forsythia libraries. The GC content of these libraries ranged from 43.99% to 46.04%. The Q30 percentage (sequencing error rates < 0.1%) of 24 libraries ranged from 92.12% to 94.05%, indicating that the quality of sequencing was sufficient for further analysis (Additional file 1). Sequence data are archived at the National Center for Biotechnology Information (NCBI SRR10829625 to SRR10829648). We mapped 88.87% to 92.87% of the clean reads to the reference genome, and more than 84.58% of them were unique mapped reads. A total of 34,352 genes were annotated by comparison with existing genomes and by blasting new genes in databases (Additional file 2).

### General patterns of transcriptomic change

**Fig. 1:**
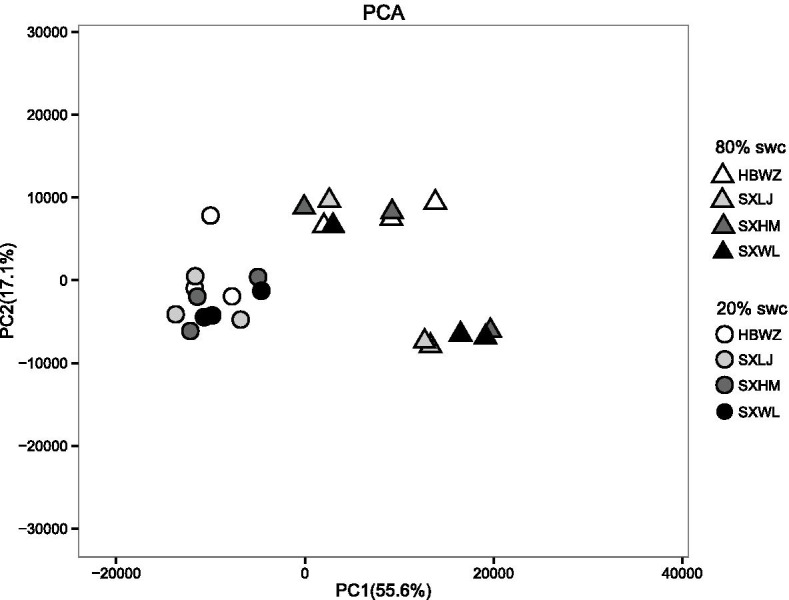
Principal component analysis of gene expression based on FPKM. Principal component 1 (PC1; 55.6% of variance) plotted against principal component 2 (PC2; 17.1% of variance). Triangles denote control samples (80% SWC) and circles denote samples exposed to drought stress (20% SWC). White symbols correspond to population HBWZ, light grey symbols to population SXLJ, dark grey symbols to population SXHM, and black symbols to population SXWL

Principal component analysis (PCA) identified major sources of variation of gene expression among all samples (Fig. 1). The PCA loading plot of gene expression indicated that drought stress was the dominant source of transcriptomic change, with weeping forsythia under 20% SWC clustering separately from those under 80% SWC (i.e., PC1; 55.6% of variance). PCA results showed that there was a considerable variation in expression among the control (80% SWC) samples, whereas only a small difference existed among the treatment group (20% SWC) samples. Although the expression among individuals was small under drought stress, the gene expression of Hebei Wuzhi Mt. (HBWZ) is closer to the upper part along PC2 axis (17.1%) as a whole than that of other three populations (Fig. 1).

**Fig. 2:**
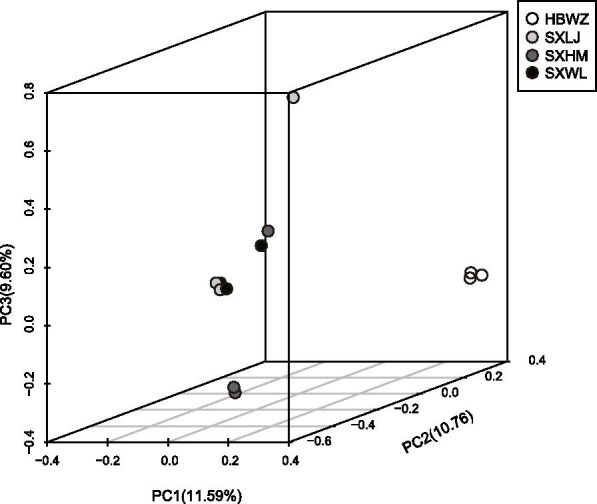
Principal component analysis of expressed gene under drought stress based on SNPs. White symbols correspond to population HBWZ, light grey symbols to population SXLJ, dark grey symbols to population SXHM, and black symbols to population SXWL

To further investigate the genetic differences in expressed genes under drought stress among the four populations, we performed another PCA analysis based on all gene SNPs (Fig. 2). These PCA results showed that the genes expressed in HBWZ under drought stress were significantly differentiated compared with those in other populations. These results indicated that HBWZ had obvious genetic differentiation from other populations on drought-related genes, which might indicate that HBWZ was different from other populations in the process of local adaptation. The results showed low correlation (*r* = 0.542, *P* > 0.05) between geographic distance and differentially expressed genes (DEGs) between populations under drought stress. There was also no low correlation (*r* = -0.466, *P* > 0.05) between geographic distance and average annual precipitation difference between populations.

### Transcriptomic response to drought stress in the four study populations

**Fig. 3:**
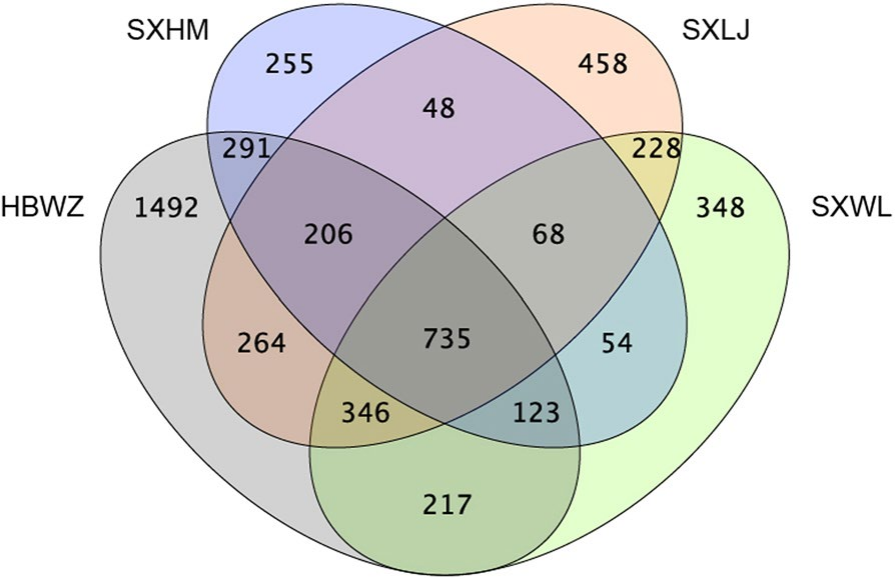
The venn diagram for DEGs of four populations

In the four surveyed populations, 3,674 DEGs in HBWZ, 1,780 in SXWL, 2,353 in SXHM, 2,119 in SXLJ underwent significant changes in abundance in response to drought stress (Fig. 3). We identified a total of 21 in HBWZ, 19 in SXWL, 15 in SXHM, 30 in SXLJ over-represented ontologies and 11 in HBWZ, 6 in SXWL, 5 in SXHM, 9 in SXLJ over-represented pathways (Additional file 3). Based on the results of these over-represented ontologies, we determined that the ontologies of all four populations related to photosynthesis, oxidation–reduction, and cell membrane component were most numerous in response to drought stress (Additional file 3). Kyoto Encyclopedia of Gene and Genome (KEGG) enriched results also showed that pathways of all four populations related to photosynthesis were most enriched (Additional file 3). In addition, the four populations share a common pathway calling carbon metabolism (Additional file 3).

### Constitutive differences in gene expression between HBWZ and other populations under drought stress

The results of DEGs under drought stress showed that the difference between HBWZ and the other three populations (HBWZ vs. SXWL: 743; HBWZ vs. SXHM: 235; HBWZ vs. SXLJ: 294) was significantly greater than that between the other three populations (SXWL vs. SXHM: 217; SXWL vs. SXLJ: 54; SXHM vs. SXLJ: 165). This result was also consistent with PCA analysis of all gene expressions, and HBWZ showed a greater difference in response to drought stress compared with the other populations. Therefore, we investigated in detail constitutive differences in gene expression between HBWZ and the other three populations under drought stress.

Based on the results of over-represented pathways and ontologies (Table 2), no significant over-represented pathways and ontologies were found between HBWZ and SXLJ. The difference between HBWZ and SXHM lies in the synthesis of aromatic substances, the synthesis of flavonoids, and oxidation–reduction process. The differences of HBWZ and SXWL in their response to drought stress mainly lie in the synthesis of flavonoids, the synthesis of aromatic amino acids, and transmembrane transport. When the *q*-value was relaxed to 0.05, we found that HBWZ was different from SXWL in the genes of aromatic substances.

**Table 2:** Representative significantly over‐represented pathways and ontologies between HBWZ and other populations under drought stress

Pathways/Ontologies	Description	*p*-value	*q*-value	Gene ID
**Constitutive differences HBWZ vs. SXWL**				
GO:0,009,813	flavonoid biosynthetic process	1.75E-05	2.90E-03	EVM0002417;EVM0002993;EVM0003393;EVM0005703;EVM0011704;EVM0014799;EVM0015905;EVM0017927;EVM0019889;EVM0026405;EVM0030102;EVM0032724
GO:0,009,073	aromatic amino acid family biosynthetic process	2.54E-05	2.90E-03	EVM0009754;EVM0012720;EVM0019603;EVM0019638;EVM0020196
GO:0,055,085	transmembrane transport	3.93E-05	2.99E-03	EVM0000891;EVM0000940;EVM0001335;EVM0002832;EVM0003592;EVM0004054;EVM0004150;EVM0005856;EVM0006591;EVM0007010;EVM0008489;EVM0010921;EVM0011770;EVM0017677;EVM0018573;EVM0019753;EVM0021320;EVM0023893;EVM0024144;EVM0024903;EVM0025722;EVM0029310;EVM0030714;EVM0032346;Forsythia_suspensa_newGene_8
GO:0,042,626	ATPase activity, coupled to transmembrane movement of substances	5.50E-07	1.30E-04	EVM0000828;EVM0000940;EVM0001335;EVM0003592;EVM0005856;EVM0010921;EVM0011770;EVM0019753;EVM0021320;EVM0025123;EVM0025722;EVM0029014;EVM0029310;EVM0032346
GO:0,016,210	naringenin-chalcone synthase activity	1.80E-05	2.13E-03	EVM0005703;EVM0015905;EVM0026405
ko00941	Flavonoid biosynthesis	1.73E-08	1.24E-06	EVM0005703;EVM0009007;EVM0015905;EVM0017927;EVM0024869;EVM0025331;EVM0025530;EVM0026405;EVM0032495;EVM0032724
**Constitutive differences HBWZ vs. SXHM**				
GO:0,055,114	oxidation–reduction process	4.61E-05	3.35E-03	EVM0000790;EVM0001940;EVM0002701;EVM0003620;EVM0005556;EVM0005614;EVM0010818;EVM0011380;EVM0012290;EVM0013140;EVM0013516;EVM0014542;EVM0014992;EVM0016163;EVM0016877;EVM0017927;EVM0019408;EVM0023577;EVM0024869;EVM0026849;EVM0028725;EVM0029461;EVM0032495;EVM0032885;Forsythia_suspensa_newGene_15854
GO:0,019,438	aromatic compound biosynthetic process	2.50E-04	9.07E-03	EVM0008181;EVM0011824;EVM0025438
GO:0,005,506	iron ion binding	2.53E-05	1.87E-03	EVM0000790;EVM0002701;EVM0003620;EVM0011380;EVM0013516;EVM0014992;EVM0015176;EVM0016877;EVM0024869;EVM0026849;EVM0032495;Forsythia_suspensa_newGene_15854
GO:0,020,037	heme binding	1.66E-04	6.11E-03	EVM0000790;EVM0002701;EVM0003620;EVM0011380;EVM0013516;EVM0014992;EVM0015817;EVM0016877;EVM0024869;EVM0026849;EVM0032495
ko00941	Flavonoid biosynthesis	4.89E-07	2.42E-05	EVM0015905;EVM0017927;EVM0024869;EVM0025530;EVM0029461;EVM0032495
ko00944	Flavone and flavonol biosynthesis	2.05E-04	5.06E-03	EVM0024869;EVM0031037
**Constitutive differences HBWZ vs. SXLJ**				
NA				

### Relationship of DEGs and sequence differentiated genes (SDGs) under drought stress among populations

Previously, SDGs were identified by searching upstream and downstream of adaptive SNPs using reduced-representation genome sequencing. Thus, we were not able to detect the genes that had sequence differentiation in the four surveyed populations. However, which pathways and ontologies are associated with heterogeneous drought stress can be detected by identifying candidate SDGs in twenty weeping forsythia populations. A total of 1,269 candidate SDGs involved into drought adaptation were identified by LFMM in previous studies [15]. Through enrichment analysis of SDGs in natural weeping forsythia populations, the ontologies related to the synthesis of aromatic substances (Table 3), such as farnesyl diphosphate metabolic process, sesquiterpene biosynthetic process, germacrene-A synthase activity, and sesquiterpene synthase activity, has undergone sequence differentiation in response to heterogeneous drought stress.

**Table 3:** Representative significantly over‐represented pathways and ontologies of sequence differentiated genes related to drought adaptation

Pathways/Ontologies	Description	Gene ID
GO:0,045,338	farnesyl diphosphate metabolic process	EVM0017569;EVM0005229;EVM0029791;EVM0003820;EVM0017781;EVM0020446;EVM0005156;EVM0032978;EVM0011395;EVM0000963;EVM0015910;EVM0032240;EVM0031429
GO:0,051,762	sesquiterpene biosynthetic process	EVM0005156;EVM0020446;EVM0017781;EVM0003820;EVM0029791;EVM0005229;EVM0017569;EVM0031429;EVM0032240;EVM0015910;EVM0000963;EVM0011395;EVM0032978
GO:0,000,287	magnesium ion binding	EVM0010166;EVM0011853;EVM0029791;EVM0020446;EVM0011351;EVM0032240;EVM0006587;EVM0000963;EVM0017781;EVM0031429;EVM0011395;EVM0018781;EVM0022374;EVM0015910;EVM0005156;EVM0017569;EVM0005229;EVM0003590
GO:0,034,005	germacrene-A synthase activity	EVM0029791;EVM0020446;EVM0005156;EVM0011395;EVM0017569;EVM0000963
GO:0,010,334	sesquiterpene synthase activity	EVM0032240;EVM0015910;EVM0031429;EVM0032978;EVM0003820;EVM0017781;EVM0005229
GO:0,008,171	O-methyltransferase activity	EVM0001095;EVM0021643;EVM0006701;EVM0006806;EVM0008181;EVM0001139;EVM0011824
ko00190	Oxidative phosphorylation	EVM0000550;EVM0001906;EVM0003509;EVM0003684;EVM0005059;EVM0006587;EVM0009269;EVM0010784;EVM0013633;EVM0016129;EVM0016416;EVM0017474;EVM0017973;EVM0019175;EVM0019658;EVM0021276;EVM0022628;EVM0024072;EVM0027979;EVM0029364;EVM0029588;EVM0032746

### Quantitative real-time transcription PCR (*q*RT-PCR) validation

**Fig. 4:**
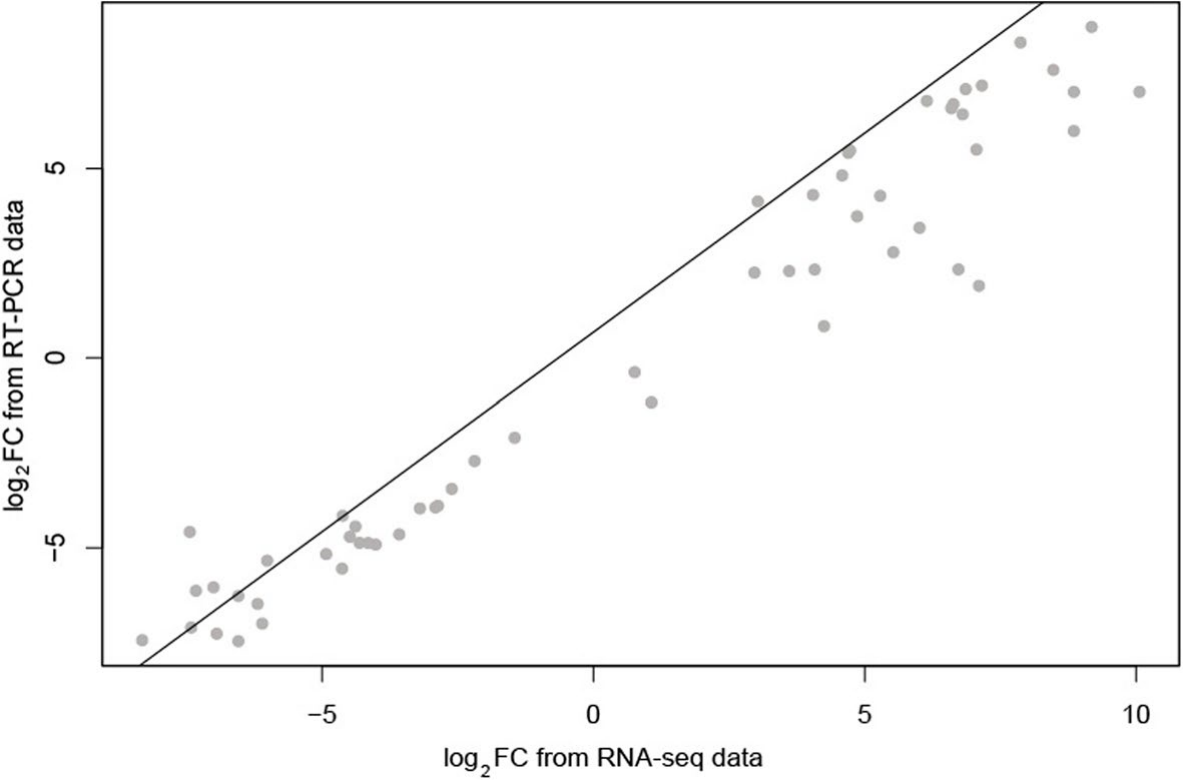
Linear correlation analysis using Pearson correlation coefficient (r) of fold change data between qRT-PCR and fold change (FC) of genes

Fourteen DEGs consisting of 7 up- and 7 down-regulated genes for drought stress were selected to confirm the reliability of the RNA-Seq results by qRT-PCR. These genes were mostly involved in transcription and posttranslational modification (Additional file 4). The expression patterns of these DEGs obtained by *q*RT-PCR were all largely consistent with the results from the RNA-seq. Significantly positive correlations (*r* = 0.972, *P* < 0.001) between the RNA-seq and *q*RT-PCR data were revealed by the Pearson correlation coefficients (Fig. 4).

## Discussion

Plants will change physiological functions through regulating gene expression when they face these environmental pressures [16]. Different populations of a species often occur in different ecological contexts, and experience adaptive differentiation in response to the local environment during over long periods of natural selection [17]. Previously, much attention has been paid to the adaptive differentiation of gene sequences among populations. The adaptive differentiation of gene sequences is stable and easily detected, which was been confirmed by many previous studies [6]. However, gene expression is more likely to be affected differentially by environmental variation. Thus, detecting the differences of gene expression among populations requires strict common garden experiments [18].

In this study, the gene transcription of four populations of weeping forsythia at 20% SWC (treatment group) were compared with those at 80% SWC (control group). Under the control conditions, no significant difference in expression profiles was found among four weeping forsythia populations, whereas significant difference in gene expression were seen among individuals (Fig. 1). This expression variation among individuals may be due to differences in genetic variation among the individuals (Figs. 2 and 3). This suggests that it is difficult to observe gene expression differences among weeping forsythia populations under normal growth conditions.

When the four populations of weeping forsythia were subjected to drought stress, however, the gene expression of the four populations was significantly different (Fig. 1). In our study, the maximum number of differentially expressed genes and metabolic pathways were found in population HBWZ. According to the annual precipitation data of these populations [15] (Additional file 5), the annual precipitation in population HBWZ was the lowest. We therefore hypothesized that population HBWZ may have evolved more mechanisms to deal with drought stress during long-term evolutionary processes. Our physiological data also supported that HBWZ might have better resistance to drought.

The gene expression differences among individuals began to shrink under drought stress, which may activate the mechanism of common resistance or response to stress. The genes differentially expressed in response to drought stress in the four populations were mostly shared. To investigate the common aspect of these four populations, enrichment analyses were performed on the DEGs of these four populations. The enrichment results of GO and KEGG both indicated that photosynthesis of all four populations was most significantly affected. Photosynthesis is highly sensitive to stress, and genes associated with it fluctuate under adverse conditions [19, 20].

In addition to photosynthesis, the four populations of weeping forsythia resist or respond to drought stress through shared genes related to oxidation–reduction, cell membrane component, and carbon metabolism. These pathways and ontologies have been confirmed to be involved in resisting or responding to drought stress in *Brassica napus* [21, 22].

**Fig. 5:**
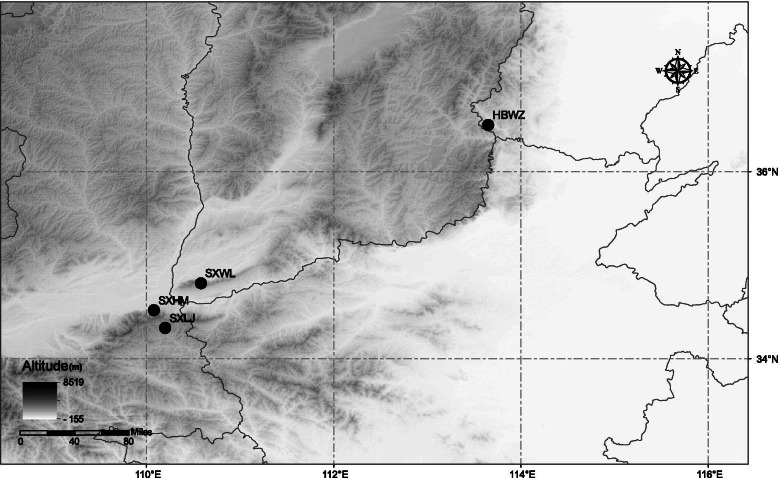
Sampling localities of 4 populations of weeping forsythia

Although the differential gene expression among individuals shrank under drought stress, we found that population HBWZ showed significant expression differentiation with the other three populations. This is confirmed by the expression of all genes (Fig. 1) and the DEGs data (Fig. 3) between all populations. However, the differences of gene expression between the three populations of SXWL, SXHM and SXLJ are obviously smaller. Taken from Fig. 5, population HBWZ is geographically distant from the other three populations, while populations of SXWL, SXHM and SXLJ are geographically close.

To verify whether the gene expression differences between populations were due to geographic distance, we performed a correlation analysis between geographic distance and the number of genes that differed between populations. The results showed that there was no significant correlation between geographic distance and the number of DEGs. There was also no low correlation (*r* = -0.466, *P* > 0.05) between geographic distance and average annual precipitation difference between populations. Therefore, we infer that differences in gene expression between these populations may be due to differences in genetic background. The PCA results based on all intragenic SNPs (Fig. 2) supported our inference that population HBWZ had significant genetic differences from the other three populations. Although there were large genetic differences among individuals in the other three populations, the genetic differences among the three populations were smaller than those between them and HBWZ.

To further investigate which genes associated with pathways and ontologies showed differential expression among the populations under drought stress, enrichment analyses were performed on DEGs between HBWZ and the other three populations. The results showed that there were 165 differentially expressed genes between the HBWZ and SXLJ populations, but they were distributed in most pathways and ontologies, and there were no significant differences in any of pathways and ontologies. However, population HBWZ showed significant differences in gene expression of flavonoids and aromatic substances with SXHM and SXWL. Flavonoids play an important role in the growth of plants, and they can help plants resist a variety of adverse environments [23]. In response to drought stress, flavonoids were shown to reduce stress symptoms in multiple species [24, 25].

Our results demonstrated that there were constitutive differences in gene expression of flavonoids among populations. Differences in flavonoid gene expression may lead to differences in flavonoid synthesis. Flavonoids has been confirmed have been proved to improve the drought tolerance of plants. For example, flavonoid synthesis gene, UDP-glycosyltransferase (EVM0002417; Additional file 6), can improve the drought tolerance of plants by regulating anthocyanin accumulation [26]. Different populations may regulate flavonoid content to cope with drought stress in the evolutionary process, and this difference was fixed even under the identical intensity of stress. The aromatic substances of plants play important roles in preventing the invasion of pathogenic bacteria, repelling pests and attracting insects for pollination [27]. However, there are few reports concerning the relationship between plant aromatic substances and stress, especially drought stress. A recent transgenic study first reported that aromatic components of plants can improve plant cold tolerance [28]. Our study suggests that aromatic substances in plants may also be related to drought tolerance, and there are differences in the synthesis of aromatic substances among different populations due to long-term drought stress with different intensities.

Besides flavonoids and aromatic substances, we also found significant differences in the genes related to oxidation–reduction process, the synthesis of aromatic amino acids, and transmembrane transport among different weeping forsythia populations. These are also fixed differences in the evolution of different populations of weeping forsythia, and the genes in these pathways have been implicated in the resistance or response to drought stress in many studies [29–31]. For example, the overexpression of the genes cytochrome P450 (EVM0000790, EVM0002701, EVM0003620; Additional file 6) in the ontology of oxidation–reduction process enhances drought stress tolerance by promoting root development [32]; overexpression of the gene of alcohol dehydrogenase (EVM0005614; Additional file 6) in *Arabidopsis* improved stress resistance to drought [33]. Therefore, we speculate that the difference in expression of the genes in oxidation–reduction process among populations may cause the difference in drought tolerance.

Another important objective of our study was to reveal the relationship between SDGs and DEGs related to drought stress among weeping forsythia populations. We reanalyzed previous study [15] and found the genes of ontologies related to the synthesis of aromatic substances such as the farnesyl diphosphate metabolic process, sesquiterpene biosynthetic process, germacrene-A synthase activity, and sesquiterpene synthase activity expressed sequence differentiation in different natural weeping forsythia populations under different intensities of drought stress.

The results of the comparative transcriptomics in this study also confirmed that genes associated with the synthesis of aromatic substances among weeping forsythia populations are also differentially expressed. According to the current and previous [15] studies, aromatic substances play important roles in the drought stress response of weeping forsythia. Populations of weeping forsythia respond to drought stress with different intensities by dual differentiation of gene sequences and expression. Interestingly, only a few genes were shared between SDGs and DEGs, and most of them are nonoverlapping.

**Fig. 6:**
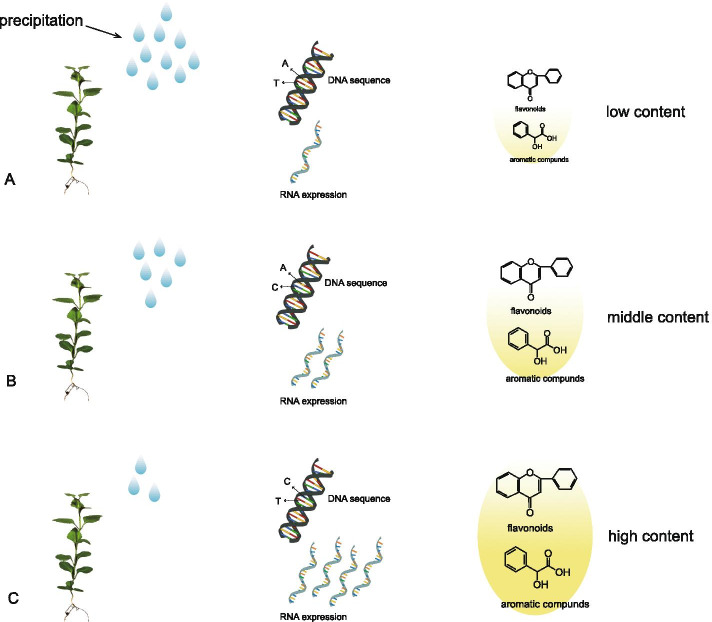
Working model for different populations of weeping forsythia involved in drought stress by regulating the content of aromatic substances and flavonoid

Our study supports the hypothesis that dual differentiation in sequence differentiation and expression occurs among different populations in response to heterogeneous environmental conditions during the process of local adaptation. This study also proposed a new working model of weeping forsythia adaptive evolution under different intensities of drought stress (Fig. 6). Under low levels of precipitation, the weeping forsythia express more genes regulating the synthesis of the aromatic substances and flavonoids, which finally lead to the accumulation of flavonoids and aromatic components under drought conditions. Thus, different populations of weeping forsythia responded to drought stress of different intensity by regulating the expression of genes related to the synthesis of flavonoids and aromatic substances, and by fixing different genotypes of the genes related to aromatic substances.

## Conclusions

In the present study, we investigated the physiological changes under drought treatment and compared the differences in gene expression among different weeping forsythia populations under drought stress in common garden experiments. Physiological results showed that HBWZ might have higher drought tolerance among four populations. RNA-seq results indicate that differential expression occurred among populations even under identical stress conditions. Significant differential expression occurred in the genes responding to the synthesis of flavonoids, aromatic substances, and aromatic amino acids, oxidation–reduction process, and transmembrane transport. This differentiation is consistent with evolution of these populations under different intensities of drought stress. Our study is the first to propose that the aromatic substances in plants might be responsible for drought tolerance. Sequence differentiation was found in the genes related to aromatic synthesis among different weeping forsythia by further reanalysis of previous study. Our study supports the hypothesis that dual differentiation in sequence differentiation and expression occurs among different populations in response to heterogeneous environmental conditions during the process of local adaptation. We also proposed a new working model of weeping forsythia evolution under different intensities of drought stress, which provides new insights for understanding the genetic mechanisms of local adaptation of species.

## Methods

### Common garden planting

Fruits of weeping forsythia were collected from four natural populations (Fig. 5, Table 4) and brought back to the laboratory for drying. Four natural populations of weeping forsythia were from Shanxi, Shaanxi and Hebei, which were the main producing areas. The provenance of artificial cultivation in these areas mainly comes from its wild resources. Seeds are harvested from the fruits from ten individuals, then mixed and randomly selected seeds for further planting. At total of 50 to 80 seeds with full grain and glossy seed coat were selected from each population. The seeds were immersed in 0.5% potassium permanganate solution for 2 h, then soaked for another 8 h in distilled water after rinsing. The soaked seeds were sown in planting pots containing nutrient soil (river sand: perlite: peat soil = 1: 2: 3) until the radicle burst the testa. The germinated seeds were then transplanted to a planting pot (9 cm × 11 cm) with one plant per pot. The seedlings were grown at 25 °C/20 °C (14 h/10 h, day/night), 20,000 lx light intensity, and 60% relative humidity in climate chamber (PLD-500-G4, Ledian Instrument Manufacturing Co., Ltd) for six months. The seedlings with good external shape and disease-free were selected for drought stress treatment.

**Table 4:** Population latitude, longitude, and precipitation of four populations in weeping forsythia

Population no. and code	Locations	Latitude (N)	Longitude (E)
1. HBWZ	Wuzhi Mt., Hebei	36.51	113.65
2. SXWL	Wulaofeng, Shanxi	34.81	110.58
3. SXHM	Hua Mt., Shaanxi	34.52	110.08
4. SXLJ	Laojun Mt., Shaanxi	34.33	110.20

### Drought treatment

According to the methods of Liu et al. [34], the seedlings from the four populations were watered to saturate the soil prior to initiating the drought treatments. Three seedlings were randomly selected from the preceding seedlings in each treatment. The experiment then sampled each population when the SWC dropped to 80% and 20%, which takes an average of five days. The leaves were sampled at three hours after SWC reached 20% SWC. Mature and robust leaves were first frozen in liquid nitrogen and then stored in a -80℃freezers. To avoid the difference at different time points caused by individual differences, we selected two time points from the same plants. The samples with 80% SWC were set as the control, and the samples with 20% SWC were set as treatment groups. The growth conditions of seedlings were set with the same conditions as above. The experimental treatments were designed with three biological replicates.

### Determination of physiological indexes

Three typical physiological indicators, Pro, SS, and MDA were determined to evaluate the drought tolerance of weeping forsythia. Pro and SS are important osmoregulation substance, their content in plants increases obviously under drought stress [35]. Thus, Pro and SS content in plants is usually used to reflect the resistance in response to drought stress. Pro and SS content were determined according to the methods of Bates et al. [36] and Rosa et al. [37], respectively. MDA content reflects the degree of peroxidation and damage to the cell membrane under drought environment. In this study, MDA content was determined by using the thiobarbital acid colorimetry method [38]. Each treatment was designed with three biological replications. All the physiological indicators were determined on microplate reader Infinite M PLEX (Tecan, Grödig, Austria). The physiological results were submitted to one-way analysis of variance followed by the least-significant difference test (*P* < 0.05) to evaluate difference among four populations, the analyses were performed in R.

### cDNA library preparation and RNA sequencing

RNA was extracted using a plant RNA Isolation kit (DP432, Tiangen Technologies, Beijing, China) following manufacturer’s instructions. RNA quality and purity were assessed using Agilent 2100 (Agilent Technologies, CA, USA) and NanoDrop One (Thermo Fisher, DE, USA). RNA with the Ratios of OD260/OD280 above 2.0 was used in the next experiment. Illumina RNA-seq libraries were created from 10 μg RNA from each sample using the NEBNext UltraTM RNA Library Prep Kit (New England BioLabs, MA, USA) according to manufacturer's instructions. Resulting libraries were further assessed using the Agilent 2100 (Agilent Technologies, CA, USA). The qualified libraries were then sequenced on an Illumina HiSeq X-ten sequencer (Illumina, CA, USA) at BioMarker Technologies (Beijing, China).

### Sequence processing and function annotation

Raw reads were filtered to remove the adaptor sequences, and low-quality bases with more than 10% anonymous nucleotides (N) and more than 50% of bases possessing a value Q ≤ 10. The remaining clean reads were mapped to the genome of weeping forsythia [15] using the software HISAT2 [39] and assembled with StringTie [40]. SNPs were called using the Haplotype Caller in GATK across all samples of *F. suspensa*. The low-quality SNPs (QUAL < 30, MQ < 40.0, FS > 60.0, and QD < 2.0) were removed. The Unigenes were annotated using the annotation information of the weeping forsythia genome [15]. New genes were annotated by comparing them with NCBI non-redundant, Swiss-Prot [41], Gene Ontology (GO) [42], the Cluster of Orthologous Group [43], evolutionary genealogy of genes: Non-supervised Orthologous Group [44], Pfam [45] and the KEGG [46] databases using BLASTX, with a significance threshold of *E* value < 10^-5^.

### Analyses of gene expression

To assess the genetic differences of expressed genes between populations, PCA was performed based on all SNPs in these genes using the plotPCA function in DESeq2 software [47]. The gene expression level of all genes in the four populations was calculated by fragments per kilobase of transcript per million fragments mapped (FPKM) using StringTie [40]. To resolve broad patterns of variation of weeping forsythia among populations in response to drought stress, PCA was performed based on FPKM value of all expressed genes using the plotPCA function in DESeq2 [40]. To identify the DEGs within and among the four populations under drought stress, DESeq2 software [47] was used with fold change ≥ 2 and a false discovery rate < 0.05 as the cut-off criteria. Here, the gene expression at the status of 80% SWC were set as the control, and the gene expression at 20% SWC were set as treatment groups. When comparing gene expression differences between populations at 20% SWC, HBWZ population were set as control. To reveal the enriched GO terms and their hierarchical position, GO enrichment analysis was implemented by the topGO packages [48] in R. The pathway enrichment analysis was used to test whether pathways are over-presentation with DEGs. Pathway significant enrichment analysis was based on pathways in the KEGG database, and the hypergeometric test was used to find pathways with significant enrichment compared with the whole genomic background. KOBAS [49] software was used to test the statistical enrichment of KEGG pathways, with *q*-values < 0.01 as the cut-off criteria. To examine the correlation between DEGs and geographic distance and average annual precipitation difference in response to drought stress among populations of weeping forsythia, correlation analysis was performed by *cor.test* in R. The average annual precipitation of four populations was from Li et al. [15]. Geographical distance was calculated by the geosphere package [50].

### qRT-PCR validation

To verify the reliability of transcriptome sequencing, quantitative real-time transcription PCR (*q*RT-PCR) was performed. Fourteen DEGs were selected, and the primers (Additional file 4) of these genes for qRT-PCR were designed using primer premier 5.0 [51]. The qRT-PCR reaction was executed using the TB Green Premix Ex Taq II (TaKaRa, Beijing, China) and carried on ABI QuantStudio®3 Real-Time System (Applied Biosystems, CA, USA). The amplification procedure was started with an initial denaturation at 95℃ for 10 min, followed by 40 cycles of 95℃ for 15 s, and 60℃ for 1 min. The *α* elongation factor [52] was used as an internal control for *q*RT-PCR amplification, and all reactions were set in triplicate. The relative 2^−△△Ct^ method was used to determine the expression levels of the 14 tested genes [53]. Pearson correlation analysis was performed between the data of qRT-PCR and transcriptome sequencing by *cor.test* in R.

### Enrichment analysis of sequence differentiated genes related to drought in previous studies

The SDGs related to drought from LFMM analysis [54] in previous studies [15] were reanalyzed. GO enrichment analysis was implemented by the topGO packages [48] with default parameters to investigate the enriched GO terms and the hierarchical position of SDGs. KEGG enrichment analysis based on the hypergeometric test was performed to find significant enrichment pathways. KEGG enrichment analysis was performed by using KOBAS software [49] with default parameters. The pathways and ontologies with *q*-values lower than 0.01 were considered significant.

## Supplementary Information

**Additional file 1.** Summary of sequence data from 24 samples.

**Additional file 2.** The annotated information of all genes.

**Additional file 3.** The GO and KEGG enrichment in four populations.

**Additional file 4.** The primers of qRT-PCR and gene function for 14 selected genes.

**Additional file 5.** The data of average annual precipitation for four populations.

**Additional file 6.** The expression and annotation information of the genes in discussion.

## Data Availability

The datasets supporting the conclusions of this article are included within the article and its additional files. Material samples are available from authors.

## Abbreviations

DEGs: Differentially expressed genes
FPKM: Fragments per kilobase of transcript per million fragments mapped
GO: Gene Ontology
HBWZ: Hebei Wuzhi Mt.
KEGG: Kyoto Encyclopedia of Gene and Genome
PCA: Principal component analysis
qRT-PCR: Quantitative real-time transcription PCR
SDGs: Sequence differentiated genes
SWC: Soil water content
SXWL: Shanxi Wulaofeng
SXHM: Shaanxi Hua Mt.
SXLJ: Shaanxi Laojun Mt.
